# Non-parametric Bayesian deep learning approach for whole-body low-dose PET reconstruction and uncertainty assessment

**DOI:** 10.1007/s11517-025-03296-z

**Published:** 2025-01-23

**Authors:** Maya Fichmann Levital, Samah Khawaled, John A. Kennedy, Moti Freiman

**Affiliations:** 1 The Interdisciplinary Program for Robotics and Autonomous Systems, Technion – Israel Institute of Technology, Haifa, Israel; 2 The Interdisciplinary Program in Applied Mathematics, Faculty of Mathematics, Technion – Israel Institute of Technology, Haifa, Israel; 3 Department of Nuclear Medicine, Rambam Health Care Campus, Haifa, Israel; 4 B. Rappaport Faculty of Medicine, Technion – Israel Institute of Technology, Haifa, Israel; 5 Faculty of Biomedical Engineering, Technion – Israel Institute of Technology, Haifa, Israel

**Keywords:** Positron emission tomography (PET), Low-dose reconstruction, Bayesian deep learning

## Abstract

**Abstract:**

Positron emission tomography (PET) imaging plays a pivotal role in oncology for the early detection of metastatic tumors and response to therapy assessment due to its high sensitivity compared to anatomical imaging modalities. The balance between image quality and radiation exposure is critical, as reducing the administered dose results in a lower signal-to-noise ratio (SNR) and information loss, which may significantly affect clinical diagnosis. Deep learning (DL) algorithms have recently made significant progress in low-dose (LD) PET reconstruction. Nevertheless, a successful clinical application requires a thorough evaluation of uncertainty to ensure informed clinical judgment. We propose NPB-LDPET, a DL-based non-parametric Bayesian framework for LD PET reconstruction and uncertainty assessment. Our framework utilizes an Adam optimizer with stochastic gradient Langevin dynamics (SGLD) to sample from the underlying posterior distribution. We employed the Ultra-low-dose PET Challenge dataset to assess our framework’s performance relative to the Monte Carlo dropout benchmark. We evaluated global reconstruction accuracy utilizing SSIM, PSNR, and NRMSE, local lesion conspicuity using mean absolute error (MAE) and local contrast, and the clinical relevance of uncertainty maps employing correlation between the uncertainty measures and the dose reduction factor (DRF). Our NPB-LDPET reconstruction method exhibits a significantly superior global reconstruction accuracy for various DRFs (paired *t*-test, $p<0.0001$, *N*=10, 631). Moreover, we demonstrate a 21% reduction in MAE (573.54 vs. 723.70, paired *t*-test, $p<0.0001$, *N*=28) and an 8.3% improvement in local lesion contrast (2.077 vs. 1.916, paired *t*-test, $p<0.0001$, N=28). Furthermore, our framework exhibits a stronger correlation between the predicted uncertainty 95th percentile score and the DRF ($r^2=0.9174$ vs. $r^2=0.6144$, *N*=10, 631). The proposed framework has the potential to improve clinical decision-making for LD PET imaging by providing a more accurate and informative reconstruction while reducing radiation exposure.

**Graphical abstract:**

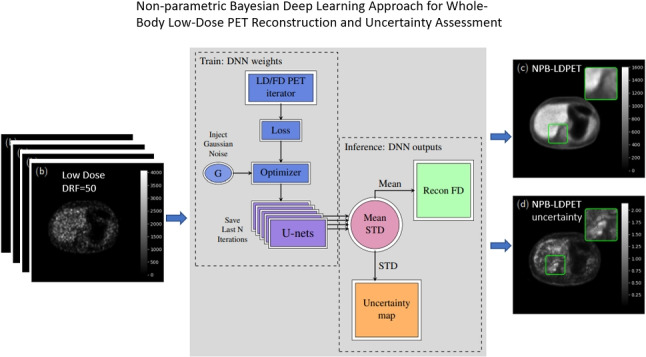

## Introduction

Positron emission tomography (PET) imaging is a vital component in managing oncological patients. For many indications, PET imaging demonstrates exceptional ability to identify tumors and track treatment-related changes with heightened sensitivity and earlier detection than conventional anatomical imaging techniques such as MRI and CT [11]. The PET system utilizes the detection of $\gamma$-ray pairs (positron annihilation photons) emanating from a radiotracer linked with a biologically active molecule administered into the body. This allows for the real-time measurement of the spatial distribution of functional processes like glucose metabolism in living tissue [10].

A key consideration in PET imaging is balancing image quality with radiation exposure or scan duration. The radiation dose from a standard PET scan can increase an individual’s lifetime cancer risk by $0.04\%$ [19], a risk that is compounded for patients requiring repeated scans. However, reducing the scan duration or radiotracer dose may compromise image quality, potentially decreasing sensitivity and leading to false negatives, particularly in detecting early-stage cancers or small metastases.

Despite advances in PET hardware, such as extended axial fields of view, decreased scanner diameters, high-performance detectors, and electronic collimation, the effective sensitivity for whole-body imaging remains limited [2]. This highlights the importance of improvements in software-based image reconstruction techniques, as hardware advancements alone cannot sufficiently address the challenges of PET imaging at low doses.

In this work, we address the challenge of clinically reliable LD PET reconstruction by introducing a non-parametric Bayesian deep learning (DL) approach for LD PET image reconstruction and uncertainty estimation. Using stochastic gradient Langevin dynamics (SGLD) [27] to sample from the posterior distribution of network weights during training, our method enhances reconstruction accuracy and provides uncertainty estimates, enabling more robust clinical interpretation. Further, SGLD-based training improves generalization, reduces overfitting, and handles non-convex problems effectively [17]. Our experiments on the ultra-low-dose PET challenge dataset [29] demonstrated improved LD PET image reconstruction, measured by the peak signal-to-noise ratio (PSNR), structural similarity index (SSIM), and normalized root mean square error (NRMSE) utilizing the FD images as a reference in comparison to the baseline Monte Carlo dropout approach [13].

We further demonstrated clinical impact by improving local lesion reconstruction accuracy, as evaluated by mean absolute error (MAE) and lesion contrast. Specifically, our contributions are as follows:

- A non-parametric Bayesian approach for low-dose PET reconstruction with uncertainty estimation.
- Uncertainty measures derived from non-parametric Ba-yesian deep learning low-dose PET reconstruction correlated with the dose reduction factor.
- Improved reconstruction accuracy and lesion conspicuity compared to conventional deep learning methods.

The remainder of this paper is organized as follows: Sect. 2 reviews related work in PET imaging, low-dose reconstruction techniques, and uncertainty estimation in deep learning. Section 3 provides the mathematical background and introduces the proposed NPB-LDPET method. Section 4 describes the evaluation methodology, and Sect. 5 presents our results. Finally, Sect. 6 concludes our work and discusses open questions and potential directions for future studies.

## Related work

Advancements in image processing and reconstruction algorithms have been instrumental in enhancing PET image quality. Analytical PET reconstruction methods, such as direct Fourier reconstruction and filtered back projection (FBP), rely on mathematical formulations based on scanner acquisition geometry [23]. Although these methods are computationally efficient, their accuracy is limited as they do not account for stochastic variability in photon detection or physical degrading factors in the acquisition system.

Advanced statistical methods, like ordered subsets expectation maximization (OSEM), incorporate physical factors but suffer from low signal-to-noise ratio (SNR) due to the ill-conditioned nature of the reconstruction problem [6, 9, 23]. Although iterative truncation mitigates noise, it often reduces contrast and SNR [20]. Filtering methods used to reduce noise typically lower spatial resolution, as traditional filters assume a linear shift invariant (LSI) system, which does not accommodate PET’s spatially variant Poisson noise characteristics.

Deep learning (DL) has recently led to substantial advancements in medical imaging [14]. Convolutional neural networks (CNNs) have shown the potential to significantly reduce PET scan dose requirements [1]. For example, recent studies have achieved dose reductions up to 200-fold using U-Net encoder-decoder architectures that incorporate multiple axial adjacent slices [28]. Chen et al. demonstrated that combining spatially correlated MRI and PET data can improve dose reduction [3]. Xue et al. developed a network based on a conditional generative adversarial network (c-GAN) to generate full-dose images from low-dose data [29]. Wang et al. combined an adversarial network with a U-Net architecture, using a task-specific perceptual loss function to classify clinical conditions accurately [26].

Despite significant progress, DL-based LD PET reconstruction methods face challenges in clinical translation, primarily due to the lack of interpretable uncertainty estimates. Providing pixel-wise uncertainty maps can aid clinicians in assessing the reliability of reconstructed images, especially in critical regions, improving interpretability and clinical relevance [24]. Bayesian DL-based models, such as variational autoencoders (VAEs) [5, 16] and Monte Carlo dropout [13], have been proposed to quantify uncertainty. Sudarshan et al. recently explored the direct prediction of uncertainty maps as part of DL models [22], though their clinical utility remains invalidated. Vlašić et al. [25] introduced a deep learning-based framework for reconstructing standard-dose PET images from low-dose PET and high-quality MRI images, accompanied by physically meaningful uncertainty quantification. Utilizing a conditional generative adversarial network (cGAN) with a noise-injection mechanism, the proposed method generates diverse posterior samples, enabling reliable uncertainty maps that improve the interpretability and potential clinical impact of PET imaging.

Recently, Sharma et al. [21] proposed a novel semi-supervised, adversarial, and variational deep neural network (DNN) framework, named Adversarial Expectation Maximization (AdvEM), for enhancing image quality in low-dose PET and CT scans. By incorporating expectation maximization, Metropolis-Hastings sampling, and adversarial training, this method enables robust image reconstruction, per-pixel uncertainty estimation, and effective learning with limited paired data. For a general review of methods for uncertainty estimation in deep learning for medical imaging, we refer the reader to the review by Huang et al. [8]. Our approach builds on these methods by incorporating non-parametric Bayesian techniques, leveraging SGLD to provide robust uncertainty estimates that enhance the clinical interpretability of LD PET reconstructions.

## Methods

### Non-parametric Bayesian DL-based PET reconstruction

The LD PET reconstruction using a deep neural network (DNN) model can be formulated as follows:

1 $$\begin{aligned} \tilde{I}_{FD}=f_{\theta }(I_{LD}) \end{aligned}$$

where $I_{LD}$ is the LD image, $I_{FD}$ is the full-dose (FD) image, and $f_\theta (I_{LD})$ is the predicted FD image from the DNN with the parameters $\theta$. The supervised training process aimed to find the DNN parameters $\theta$ is formulated as follows:

2 $$\begin{aligned} \widehat{\theta } =\underset{\theta }{\operatorname {argmin}} L\left( f_{\theta }(\mathcal {I_{LD}}),\mathcal {I_{FD}}\right) \end{aligned}$$

where *L* is the loss function, and $\mathcal {I_{LD}}=\left\{ I_{LD}^0, \ldots , I_{LD}^N\right\}$, $\mathcal {I_{FD}}=\left\{ I_{FD}^0, \ldots , I_{FD}^N\right\}$ are the sets of the corresponding low-dose and full-dose images.

From a probabilistic point of view, this formulation of the DNN training process yields a maximum likelihood point estimate of the DNN parameters. This may result in a potential overfitting that yields sub-optimal results at the inference phase. Further, it lacks the ability to quantify the uncertainty estimate of the reconstructed image.

To overcome these challenges, we adopt a Bayesian approach to characterize the full posterior distribution of the network parameters:

3 $$\begin{aligned}&P(\varTheta |\mathcal {I_{LD}},\mathcal {I_{FD}})\nonumber \\  &\quad = \frac{P(\mathcal {I_{LD}},\mathcal {I_{FD}}|\varTheta )}{\int _{{\varTheta }'} P(\mathcal {I_{LD}},\mathcal {I_{FD}}|{\varTheta }')P({\varTheta }')d({\varTheta }')}P(\varTheta ) \end{aligned}$$

where $P(\varTheta | I_{LD}, I_{FD})$ represents the posterior distribution of the network weights $\varTheta$ given the low-dose ($I_{LD}$) and full-dose ($I_{FD}$) PET images, encapsulating the probability distribution over all possible weight values conditioned on the observed data. The term $P(I_{LD}, I_{FD} | \varTheta )$ denotes the likelihood, measuring how well a particular set of weights $\varTheta$ predicts the observed data; higher values indicate weights that more effectively reconstruct the input. The prior $P(\varTheta )$ encodes any pre-existing assumptions about the weights, such as smoothness or sparsity, which help regularize the reconstruction to control for noise. This is particularly important for low-dose PET imaging, where assumptions can enhance image quality by reducing variability. The denominator, $\int _{\varTheta '} P(I_{LD}, I_{FD} | \varTheta ') \, P(\varTheta ') \, d\varTheta '$, normalizes the posterior to ensure it forms a valid probability distribution.

However, calculating this integral is generally intractable, making it impractical for direct use in reconstructing images from low-dose data. Previous works proposed a domain-specific formulation of the prior $P(\varTheta )$ to achieve a maximum posterior estimation in a computationally feasible time:

4 $$\begin{aligned} \widehat{\theta }=\underset{\theta }{\operatorname {argmax}}P(\varTheta |\mathcal {I_{LD}},\mathcal {I_{FD}})\propto P(\mathcal {I_{LD}},\mathcal {I_{FD}}|\varTheta )P(\varTheta ) \end{aligned}$$

where $\theta = \arg \max _{\theta } P(\varTheta | I_{LD}, I_{FD}) \propto P(I_{LD}, I_{FD} | \varTheta ) P$ $(\varTheta )$ represents the maximum a posteriori (MAP) estimate of the network parameters $\theta$. In this formulation, the MAP estimate is obtained by maximizing the posterior distribution of the weights $\varTheta$, balancing the likelihood $P(I_{LD}, I_{FD} | \varTheta )$, which measures how well the model reconstructs the data, and the prior $P(\varTheta )$, which regularizes the weights based on pre-defined assumptions, such as smoothness or sparsity. This approach yields a single set of optimized weights for reconstructing full-dose PET images from low-dose inputs, making the process computationally feasible while incorporating prior information to improve stability and reduce noise. A specific example of such a pre-defined prior can be a smoothness or sparsity term over the reconstructed image. While this can provide a useful point estimate, it does not capture the full posterior distribution, limiting its ability to quantify uncertainty in the reconstructed images.

To address this challenge, we propose to employ the SGLD approach [15, 27] during the DNN training to efficiently characterize the entire posterior distribution of the DNN weights and to achieve uncertainty-aware low-dose PET reconstruction. SGLD combines gradient-based optimization with stochastic noise, enabling the model to approximate Bayesian inference by sampling from the posterior distribution of the weights rather than converging to a single solution. By introducing controlled randomness into the training process, SGLD allows the model to explore different configurations of the weights that are consistent with the observed data, effectively capturing a range of plausible reconstructions.

This sampling-based approach provides a way to quantify the uncertainty associated with each prediction, as multiple plausible outcomes are generated based on the underlying data variability. In the context of low-dose PET reconstruction, this means that SGLD can highlight regions in the image where the model’s predictions are less certain, typically due to lower signal quality or reduced dose. By capturing the full posterior distribution, SGLD enables a robust and interpretable reconstruction process that informs clinicians about areas of high and low confidence in the reconstructed images, enhancing the clinical utility of the model by making uncertainty explicit in each pixel of the reconstruction.

**Fig. 1:**
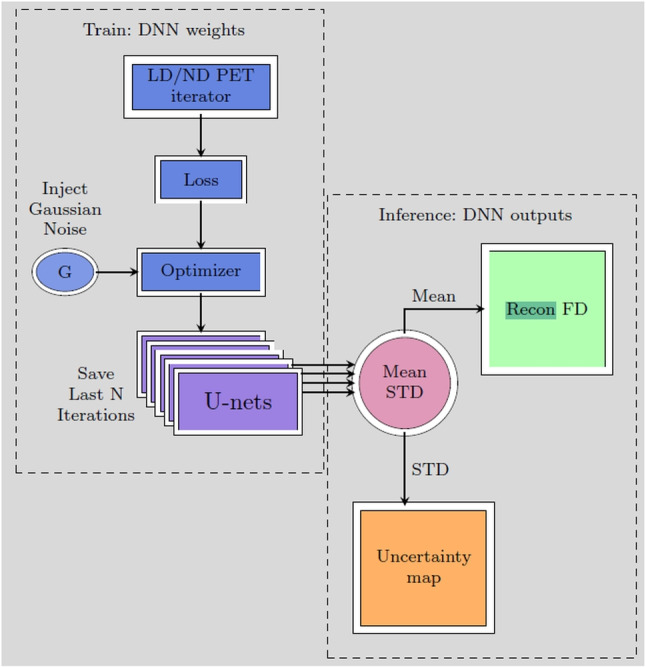
Block diagram of the proposed NPB-LDPET DNN LD reconstruction system. At the inference phase, we use a set of U-Net models with the same backbone network but different weights, which are obtained as a result of the SGLD-based training. These models predict a set of reconstructed images. The average of these images is utilized as the final reconstructed prediction, while the pixel-wise standard deviation (std.) is used to quantify for uncertainty of the predictions

Figure 1 illustrates the proposed NPB-LDPET framework. We treat the weights of the network as random variables and aim to obtain samples from the posterior distribution of the model’s prediction. To achieve this objective, we introduce a noise scheduler that injects Gaussian noise that varies over time into the loss gradients during the optimization process. Specifically, at each iteration of the training process, we incorporate Gaussian noise into the loss gradients and subsequently update the weights according to these “noisy” gradients in the next iteration.

The integration of the Gaussian noise scheduler plays a crucial role in enhancing the robustness and reliability of our model’s reconstructions. By injecting controlled Gaussian noise into the training process, the scheduler facilitates more effective exploration of the weight space, which contributes to improved posterior sampling and uncertainty estimation. This added noise helps the model generalize better across varying acquisition conditions, as it simulates variability similar to that encountered in real low-dose PET imaging environments. Consequently, the Gaussian noise scheduler aids in preventing overfitting, particularly in regions with lower signal-to-noise ratios, and leads to more stable reconstructions that are less sensitive to input variations.

We utilized an Adam optimizer for the weights update. We denote the loss gradients by the following:

5 $$\begin{aligned} g^t\overset{\triangle }{=}\nabla _{\theta }L^t(f_{\theta }(\mathcal {I_{LD}}),\mathcal {I_{FD}}) \end{aligned}$$

where $L^{t}$ is the reconstruction loss at the training iteration (epoch) *t*. At each training iteration, Gaussian noise is added to *g*:

6 $$\begin{aligned} \tilde{g}^{t}\leftarrow g^{t}+{\textbf {e}}^{t} \end{aligned}$$

where ${\textbf {e}}^{t}\sim \mathcal {N}(0,s^t)$, $s^t$ controls the noise variance. Finally, the network weights obtained during iterations $t\in \left[ {N_{T}-N+1},N_T\right]$ are saved, where $N_T$ denotes the total number of iterations and *N* represents the number of iterations aggregated after the loss curve converges. For each iteration, we set $s^t$ to be equal to the Adam learning rate.

During the inference phase, we exploit the network weights that were obtained in the last *N* iterations, $\left\{ \theta ^{t}\right\} _{N_T-N+1}^{N_T}$. We sample a set of reconstructed images $\left\{ \tilde{I}_{FD}\right\} _{N_T-N+1}^{N}$, obtained by feed-forwarding the under-sampled LD image $I_{LD}$ to the reconstruction models with the weights $\left\{ \theta ^{t}\right\} _{N_T-N+1}^{N_T}$. Then, we estimate the averaged posterior image:

7 $$\begin{aligned} \hat{I}_{FD}=\frac{1}{N}\sum _{N_T-N+1}^{N_T}{\tilde{I}_{{FD}_i} } \end{aligned}$$

We calculate the standard deviation of the reconstruction, which is utilized to assess the level of uncertainty.

Algorithm 1 summarizes our proposed approach for the training and inference phases.

**Algorithm 1:**
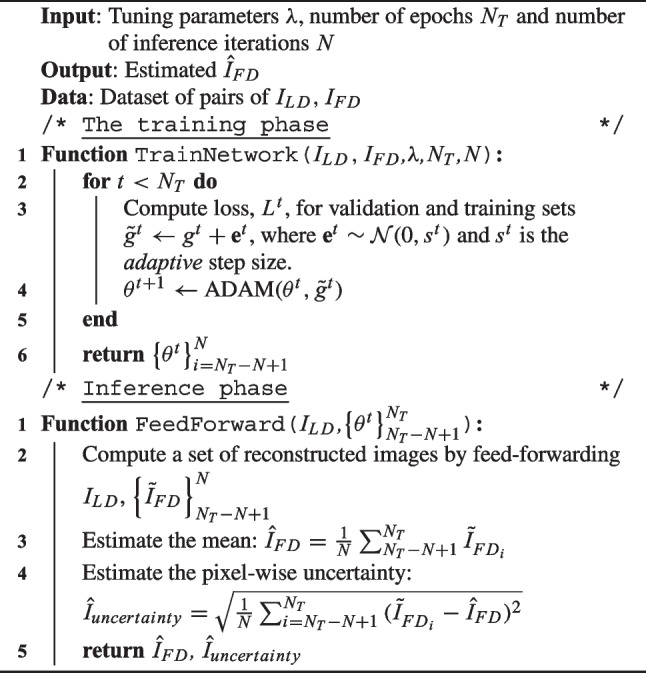
NPB-LDPET Algorithm

**Fig. 2:**
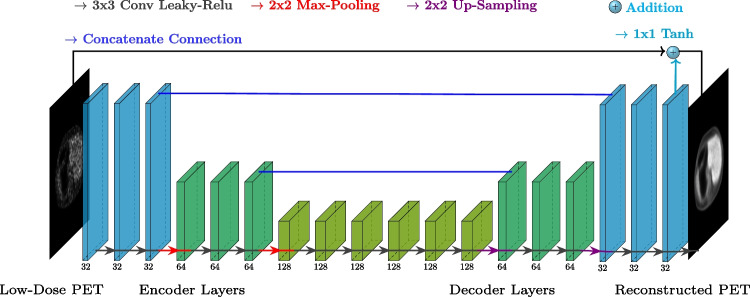
Network architecture. The proposed NPB-LDPET reconstruction system employs a U-Net architecture, where CNN layers of the same level are highlighted in the same color. The input to the network is the low-dose PET image, which is propagated through the U-Net and added to the output of the last layer to predict the reconstructed PET image

### Network architecture

Our backbone model is based on U-Net architecture. We adapted the model provided by The MONAI Consortium [4], to the image reconstruction task. Figure 2 illustrates the network architecture. The U-Net levels include $3\times 3$ convolutional layers followed by a leaky rectified linear unit (Leaky ReLU). The encoder consists of three convolutional layers with kernels of $3\times 3$ and hidden sizes of 32, 64, and 128, respectively. Max pooling of $2\times 2$ is done on the output of each layer before feeding it into the next layer. The decoder is symmetric to the encoder, with input being a concatenation of the up-sampled output of the previous layer and the corresponding encoder level. The final decoder layer output undergoes a $1\times 1$ convolution with hyperbolic tangent activation and is added to the original input for creating the output image.

### Loss function

Our loss function is a weighted combination of an $L_1$ loss and a gradient magnitude term.

The $L_1$ loss, also known as mean absolute error, is defined as follows:

8 $$\begin{aligned} L_{1}&= L_{1}(f_{\theta }(I_{LD}),I_{FD})\nonumber \\  &=\frac{1}{NM}\sum _{i=1}^N\sum _{j=1}^M|{f_{\theta }(I_{LD})}_{ij} - (I_{FD})_{ij}| \end{aligned}$$

where *N* and *M* are the number of rows and columns of the image, respectively. The $L_1$ loss is known to be superior to $L_2$ loss for image restoration tasks [30]. This is attributed to its reduced sensitivity to outliers in the reconstruction and its tendency to produce less over-smoothed images compared to the $L_2$ norm. This helps to emphasize informative structures in the image and detect subtle features or abnormalities. This is particularly important in LD PET image reconstruction, where the low SNR of the LD input requires the efficient noise reduction and blurriness reduction provided by the $L_1$ loss [26].

The gradient magnitude loss term penalizes the difference between the magnitude of the gradients of the reconstructed image and the original FD image:

9 $$\begin{aligned} {L_{1}}_{gm} = L_{1}(\left\| \nabla _{x,y} f_{\theta }(I_{LD}) \right\| _{2}, \left\| \nabla _{x,y} I_{FD} \right\| _{2}) \end{aligned}$$

where $$\left\| \nabla _{x,y} \right\| _{2} = \sqrt{\nabla _{x}^{2} +\nabla _{y}^{2}}$$

This loss has been shown to capture image structures without sacrificing the recovery of low-frequency content and is particularly useful for preserving edges and details in reconstructed images [7]. Compared to other loss functions, such as $L_2$ loss, the gradient magnitude loss can produce sharper and more accurate images, especially in regions with high activity gradients. Moreover, the use of gradient magnitude loss can reduce noise in the reconstructed image, which is beneficial for LD PET imaging applications with low SNR.

### Training details

We trained our model for 200 iterations, using an Adam [12] optimizer with a weight decay of $1e-10$ and an initial learning rate of $5e-4$, utilizing a reduced learning rate on a Plateau scheduler [18] with default parameters (reduction factor of 0.1 and patience of 10 epochs). The training was conducted with a batch size of 64 to ensure efficient use of computational resources and stable convergence. All experiments were implemented in PyTorch (version 1.8) and run on an NVIDIA Tesla V100 GPU with 32GB of RAM, providing the necessary computational power to handle high data throughput and large model parameters. This configuration allowed for efficient training times and consistent performance across all experimental runs.

During each training iteration of the SGLD-based U-Net training, we injected zero-mean Gaussian noise with a standard deviation (std.) that was set to be equal to the scheduler learning rate. We saved the network parameters that were obtained in the last *N* iterations during the training phase.

**Fig. 3:**
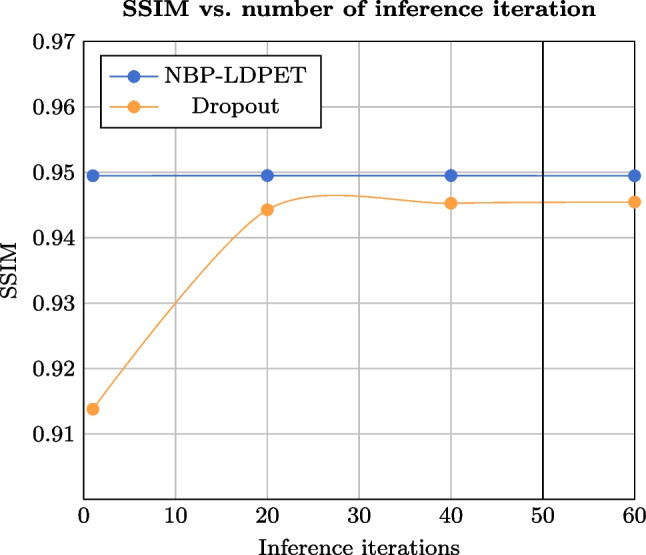
Hyper-parameters optimization results. SSIM metric, measured on the evaluation set is shown for both NBP-LDPET and dropout Monte Carlo methods and varied values of the iterations number, *N*. The results indicate that the optimal value of iterations is $N=50$, which is used in the remaining experiments

The Adam optimizer was chosen for its computational efficiency and adaptive learning rate, which are well-suited for the gradient-based sampling used in SGLD. Adam’s ability to adjust step sizes per parameter enhances stability and accelerates convergence, even with noisy gradients. This adaptability improves posterior sampling consistency, leading to more accurate reconstructions and reliable uncertainty estimation in low-dose PET images.

### Inference

At the inference stage, we input the LD images into our model, using the network parameters obtained from the last $N$ iterations of the training process to generate $N$ samples of the FD image predictions. Through hyper-parameter optimization, we determined that $N = 50$ provides a balance between computational efficiency and robust uncertainty estimation, as it captures sufficient variability in the posterior distribution without excessive computational load. The final FD output is then estimated by averaging these $N$ samples, creating a single composite image that reflects the most likely reconstruction. The standard deviation across these samples is calculated to produce a pixel-wise uncertainty map, which highlights regions where the model’s predictions are less certain.

**Fig. 4:**
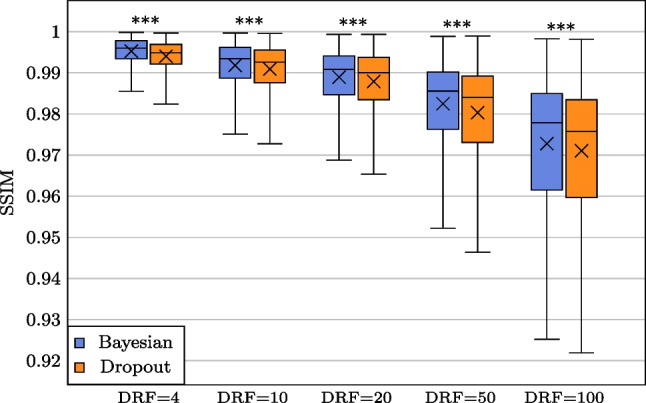
**SSIM**$\uparrow$ **box-plot for non-parametric Bayesian LD PET reconstruction (NPB-LDPET) vs. dropout method with DRFs =** 4**,** 10**,** 20**,** 50, **and** 100**.** The NPB-LDPET reconstruction approach demonstrates a statistically significant increase in SSIM which indicates superior reconstruction accuracy ($p<0.0001$)

**Fig. 5:**
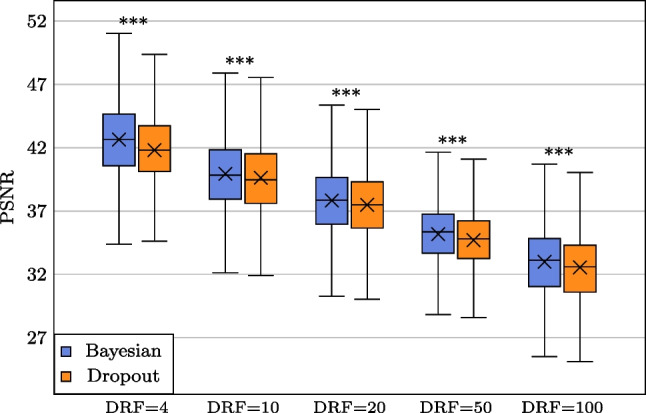
**PSNR**$\uparrow$ **scores box-plot for non-parametric Bayesian LD PET reconstruction (NPB-LDPET) vs. dropout method with DRFs =** 4**,** 10**,** 20**,** 50, **and** 100**.** The NPB-LDPET reconstruction approach demonstrates a statistically significant improved PSNR which indicates superior reconstruction accuracy ($p<0.0001$)

## Evaluation methodology

### Dataset

We used a subset of the ultra-low-dose PET challenge dataset [29] for training, testing, and validating the model performance. All images were acquired by United Imaging uExplorer ($n=330$) in list mode allowing for the rebinning of data to simulate different acquisition times. Image pairs utilized to train, test, and validate the network performance were the FD image paired with LD image with DRFs at 4, 10, 20, 50, and 100. The dataset was divided into training, validation, and evaluation subsets. Subsets of 53, 155, 10, 631, and 10, 467 image pairs were randomly selected for training, validation, and evaluation, respectively.

**Fig. 6:**
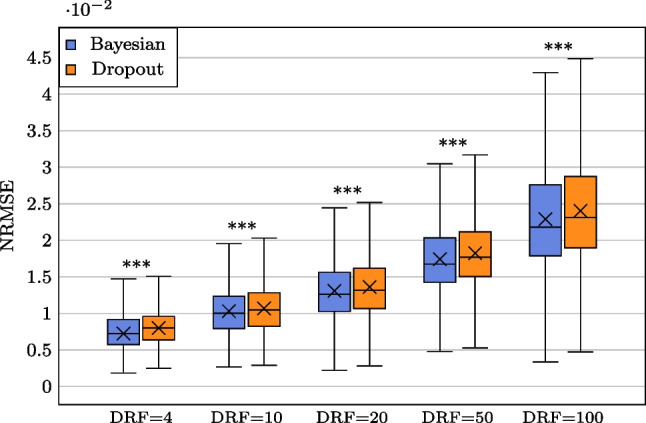
**NRMSE**$\downarrow$ **box-plot for non-parametric Bayesian LD PET reconstruction (NPB-LDPET) vs. dropout method with DRFs =** 4**,** 10**,** 20**,** 50, **and** 100**.** The NPB-LDPET reconstruction approach demonstrates a statistically significant decrease in NRMSE which indicates superior reconstruction accuracy ($p<0.0001$)

**Table 1:** Paired *t*-test SSIM$\uparrow$ scores NPB-LDPET vs. dropout DRF = 4, 10, 20, 50, and 100

SSIM NPB-LDPET vs. Dropout					
DRF	Group	*N*	Mean	Std.	*p*-value
4	NPB-LDPET	2107	**0**.**9935**	0.0106	.000***
	Dropout	2107	0.9905	0.0302	
10	NPB-LDPET	2107	**0**.**9901**	0.0109	.000***
	Dropout	2107	0.9874	0.0303	
20	NPB-LDPET	2095	**0**.**9877**	0.001	.000***
	Dropout	2095	0.9849	0.0294	
50	NPB-LDPET	2083	**0**.**9813**	0.0127	.000***
	Dropout	2083	0.9788	0.0195	
100	NPB-LDPET	2075	**0**.**9720**	0.0171	.000***
	Dropout	2075	0.9697	0.0205	

$$^{***}p<.001, ^{**}p<.01, ^{*}p<.05$$

### Benchmark testing

We used the Monte Carlo dropout Bayesian deep learning method [13] as a benchmark. We used the same U-Net architecture while adding dropout layers of probability 0.2. The Monte Carlo dropout model was trained by the same number of iterations, hyper-parameters, and training settings as the NBP-LDPET model. The final dropout model obtained during the training phase was saved for the dropout inference phase. In this phase, the LD image was fed to the Dropout model *N* times, and the final reconstructed image was calculated as the mean of the images. The uncertainty map was then determined as the std. of the images.

**Table 2:** Paired *t*-test PSNR$\uparrow$ scores NPB-LDPET vs. dropout DRF = 4, 10, 20, 50, and 100

PSNR NPB-LDPET vs. dropout					
DRF	Group	*N*	Mean	Std.	*p*-value
4	NPB-LDPET	2107	**42**.**7574**	3.4462	.000***
	Dropout	2107	41.937	3.1209	
10	NPB-LDPET	2107	**40**.**0977**	3.3002	.000***
	Dropout	2107	39.7389	3.3772	
20	NPB-LDPET	2095	**37**.**9274**	3.1907	.000***
	Dropout	2095	37.5502	3.2118	
50	NPB-LDPET	2083	**35**.**3297**	3.0274	.000***
	Dropout	2083	34.8829	3.0422	
100	NPB-LDPET	2075	**33**.**1189**	2.9613	.000***
	Dropout	2075	32.6789	3.014	

$$^{***} p<.001, ^{**} p<.01, ^{*} p<.05$$

**Table 3:** Paired *t*-test NRMSE$\downarrow$ scores NPB-LDPET vs. dropout DRF = 4, 10, 20, 50, and 100

NRMSE NPB-LDPET vs. dropout					
DRF	Group	*N*	Mean	Std.	*p*-value
4	NPB-LDPET	2107	**0**.**0079**	0.0033	.000***
	Dropout	2107	0.0085	0.0033	
10	NPB-LDPET	2107	**0**.**0106**	0.004	.000***
	Dropout	2107	0.011	0.004	
20	NPB-LDPET	2095	**0**.**0136**	0.005	.000***
	Dropout	2095	0.0142	0.0053	
50	NPB-LDPET	2083	**0**.**0182**	0.0064	.000***
	Dropout	2083	0.0191	0.0067	
100	NPB-LDPET	2075	**0**.**0233**	0.0077	.000***
	Dropout	2075	0.0246	0.0083	

$$^{***}p<.001, ^{**}p<.01, ^{*}p<.05$$

### Hyper-parameters optimization

We optimized *N*, the number of samples used during inference, by performing internal evaluation using the training set. We divided the training data into 27, 388 training images and 6847 validation images with $DRF=50$. We trained the model for 150 iterations. Subsequently, we conducted multiple inference trials on an evaluation set of 1087 images. We varied the number of iterations used for inference ($N = 1, 20, 40, 60$) and tested the resulting SSIM. Figure 3 summarizes our hyper-parameter optimization results. As the number of iterations increases, the SSIM measure for the reconstructed images using dropout increases until it plateaus at around 40 to 60 iterations. On the other hand, the SSIM measure for the reconstructed images using NBP-LDPET remains stable as the number of iterations increases, which suggests that the model has converged. Ultimately, we decided to use $N=50$ iterations for the inference phase.

**Fig. 7:**
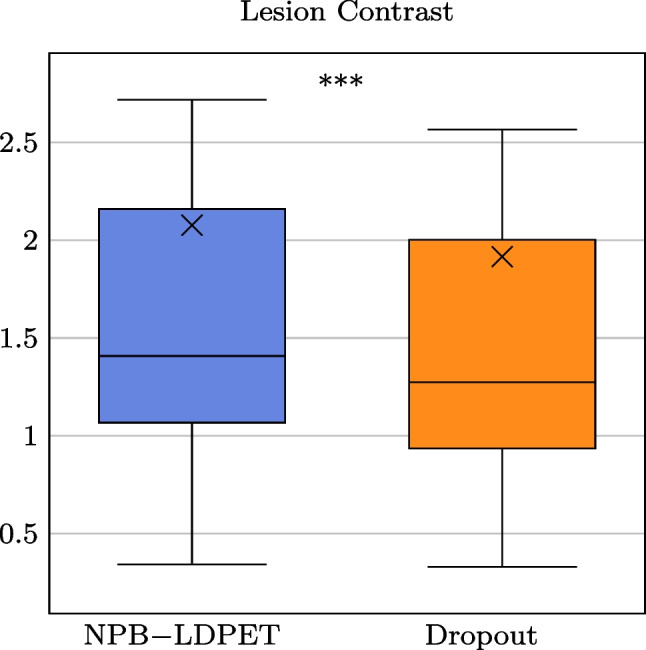
NPB-LDPET vs. dropout Monte Carlo lesion Weber contrast evaluation results. The NPB-LDPET reconstruction yields significantly better visual outcomes in lesion areas compared to dropout reconstruction (2.077 vs. 1.916, paired *t*-test $p<0.0001$, $N=28$)

**Table 4:** Paired *t*-test lesion contrast$\uparrow$ scores NPB-LDPET vs. dropout

NPB-LDPET vs. dropout					
DRF	Group	*N*	Mean	Std.	*p*-value
20	NPB-LDPET	28	**2**.**077**	1.944	.000***
	Dropout	28	1.917	1.88	

$$^{***}p<.001, ^{**}p<.01, ^{*}p<.05$$

### Evaluation

#### Reconstruction accuracy

For evaluating reconstruction quality, we computed the PSNR, SSIM, and NRMSE using the reference FD images. PSNR measures the ratio of the maximum possible signal power to the noise power, providing a quantitative indication of the fidelity of the reconstructed images relative to the FD reference. SSIM assesses perceptual similarity by comparing structural details, luminance, and contrast between the reconstructed and FD images, offering a more robust measure of visual similarity. NRMSE calculates the normalized difference between the reconstructed and reference images, allowing for an assessment of the error magnitude.

We reported these metrics for the entire evaluation set (*N*=10, 467) at DRFs of 4, 10, 20, 50, and 100. We used a paired *t*-test with $p<0.05$ to assess the statistical significance between our NPB-LDPET and dropout Monte Carlo approach.

**Fig. 8:**
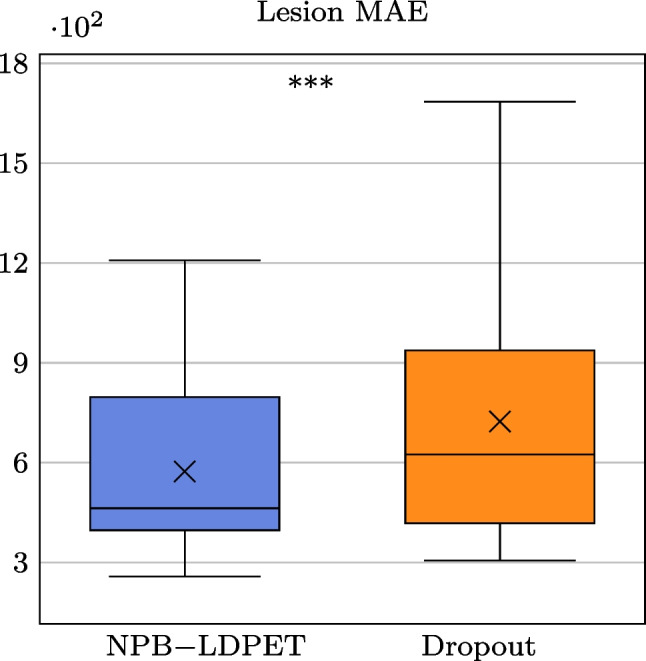
Non-parametric Bayesian (NPB-LDPET) vs. dropout lesion mean absolute error evaluation results indicate that NPB-LDPET achieves $21\%$ superior accuracy in lesion quantitation compared to dropout reconstruction method (573.54 vs. 723.70, $p<0.0001$)

**Table 5:** Paired *t*-test MAE$\downarrow$ scores NPB-LDPET vs. dropout

NPB-LDPET vs. Dropout					
DRF	Group	*N*	Mean	Std.	*p*-value
20	NPB-LDPET	28	**573**.**546**	255.323	.000***
	Dropout	28	723.706	353.146	

$$^{***}p<.001, ^{**}p<.01, ^{*}p<.05$$

**Fig. 9:**
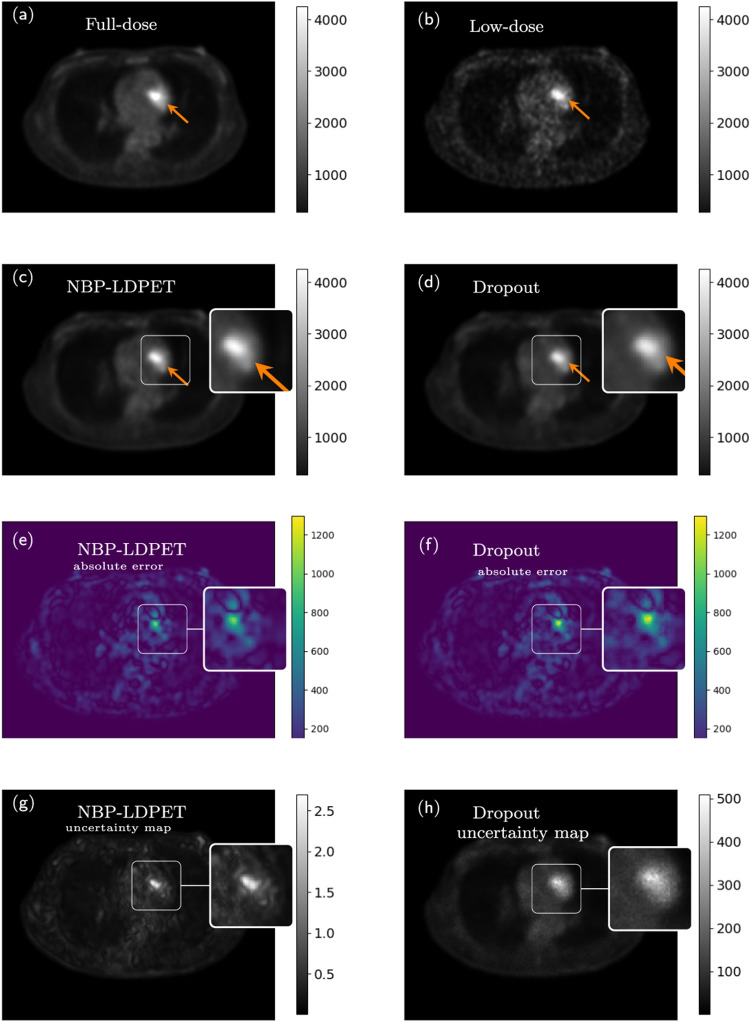
NPB-LDPET vs. dropout Monte Carlo reconstructed images for lesion ROIs. **a** Full-dose image, **b**  low-dose image at DRF=20, and **c** NPB-LDPET show superior contrast of 2.29 compared to **d**  dropout Monte Carlo reconstruction with 2.11. **e**  Lesion MAE of NPB-LDPET is $19.85\%$ smaller than **f**  dropout Monte Carlo MAE (450.54 vs. 562.18). **g**  NPB-LDPET uncertainty map depicts the lesion area more clearly and is more consistent with the absolute error map compared to **h**  dropout Monte Carlo uncertainty map

**Fig. 10:**
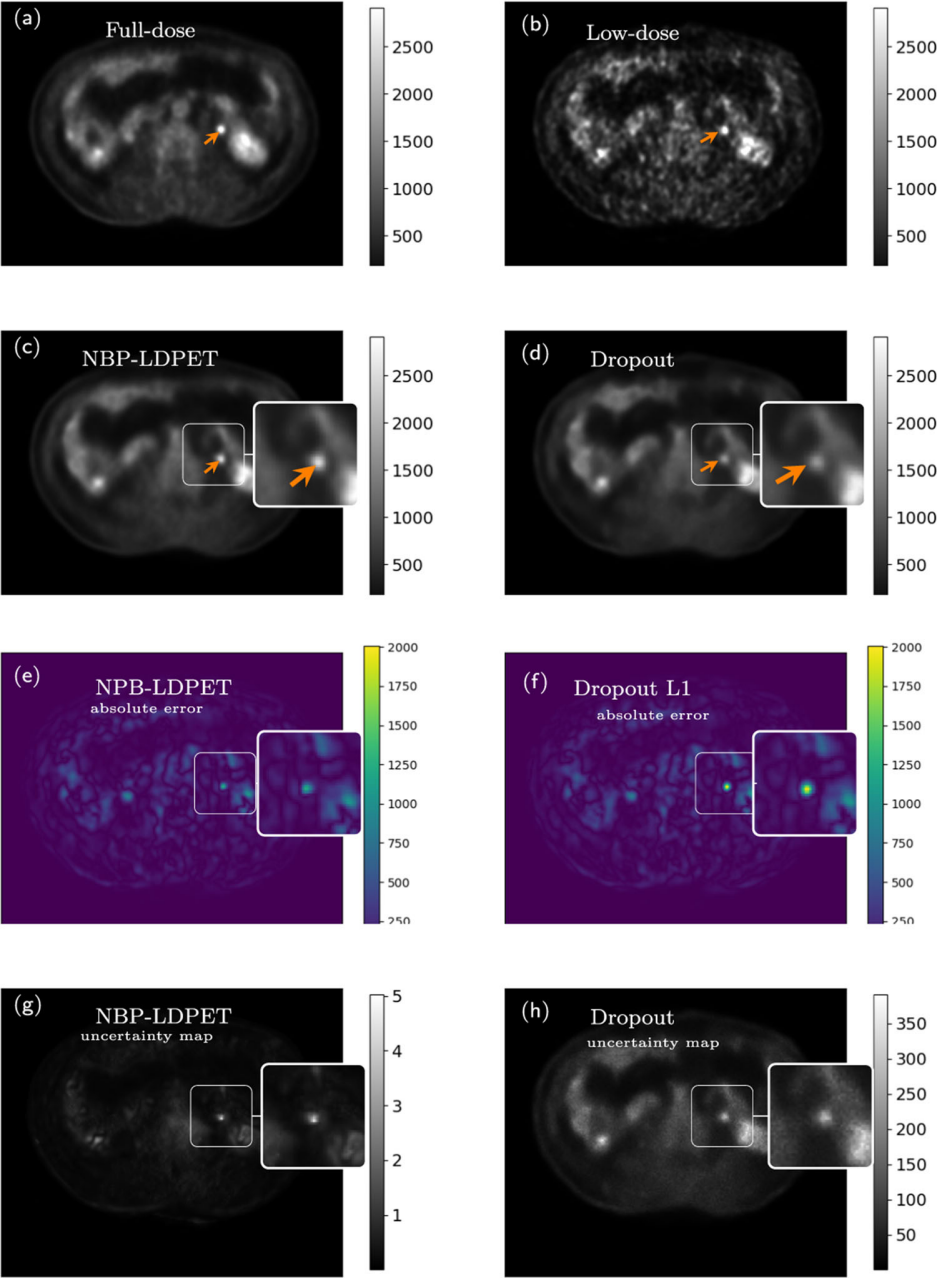
NPB-LDPET vs. dropout Monte Carlo reconstructed images for lesion ROIs. **a**  Full-dose image, **b**  low-dose image at DRF=20, **c**  NPB-LDPET show superior contrast of 1.58 compared to **d**  dropout Monte Carlo reconstruction with 0.98. **e**  Lesion MAE of NPB-LDPET is $45.05\%$ smaller than **f**  dropout Monte Carlo MAE (820.024 vs. 450.54). **g**  NPB-LDPET uncertainty map depicts the lesion area more clearly and is more consistent with the absolute error map compared to **h**  dropout Monte Carlo uncertainty map

**Fig. 11:**
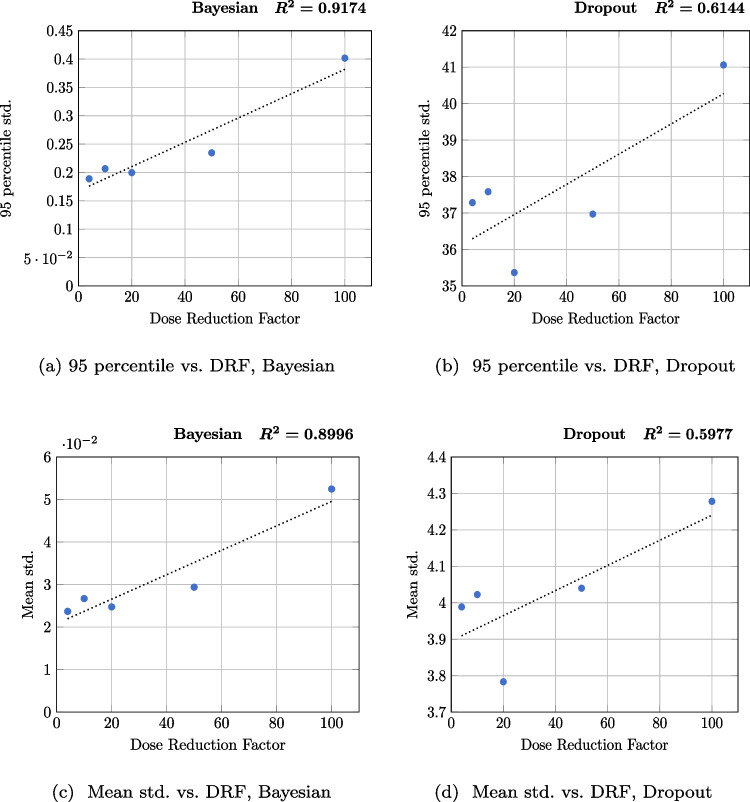
The correlation between the uncertainty score and DRF for NPB-LDPET and Dropout Monte Carlo. **a** 95 percentile std. score shows a strong correlation for the NPB-LDPET approach: $r^2$=0.9174 vs. **b** 0.6144 for the dropout Monte Carlo. This correlation is validated also by the mean std. score: **c** $r^2$=0.8996 vs. **d** 0.5977

#### Clinical impact

We assessed the clinical relevance of our approach by comparing the reconstruction accuracy (MAE) and lesion conspicuity and Weber Contrast on specific regions corresponding to pathological lesions in the images. We annotated regions of interest (ROIs) corresponding to lesions and their surrounding background marked on a total of 28 lesions marked on FD images. We projected these ROIs for analysis onto the LD images ($DRF=20$) reconstructed with our NPB-LDPET approach and with the benchmark dropout Monte Carlo.

#### Uncertainty map evaluation

We assessed the clinical relevance of our uncertainty maps by examining the correlation between the uncertainty score and DRF. Our assumption is that increased DRF should be associated with an increased uncertainty score. We computed the uncertainty maps using our NPB-LDPET approach and using the benchmark dropout Monte Carlo. We computed two distinct uncertainty map scores for each DRF: (a) the median of the 95th percentile measure of the uncertainty maps, indicating regions with high uncertainty, and (b) the median of the mean measure of the uncertainty maps, indicating overall global uncertainty. We used the $r^2$ of a linear fit as the evaluation metric.

## Results

### Reconstruction accuracy

Figures 4, 5, and 6 and Tables 1, 2, and  3 display the global evaluation metrics for our NPB-LDPET reconstruction and Monte Carlo dropout method over the entire evaluation subset ($N=10,467$ images) at DRFs of 4, 10, 20, 50, and 100.

The NPB-LD PET reconstruction outperforms the dropout Monte Carlo (paired *t*-test, $p<0.0001$) by means of SSIM, PSNR, and NRMSE for all DRFs.

### Clinical impact

Figures 9 and 10 present representative examples of the reconstructed LD images with zoom-in to the lesions. The NPB-LDPET reveals a superior contrast in and lower MAE. Furthermore, the lesion areas are highlighted with greater clarity in the uncertainty map of the NPB-LDPET method.

Figure 7 and Table 4 summarize the lesions’ conspicuity assessment results. The NPB-LDPET reconstruction approach exhibited significantly superior local lesion conspicuity by means of Weber contrast compared to the dropout Monte Carlo method (2.077 vs. 1.917, paired *t*-test with, $p<0.0001$, $N=28$).

Figure 8 and Table 5 present the lesions reconstruction accuracy results. Our NPB-LDPET method reduced the MAE of local lesions by $21\%$ compared to the dropout Monte Carlo method (573.54 vs. 723.70, paired *t*-test, $p<0.0001$, $N=28$) (Figs. 9, 10, 11).

**Fig. 12:**
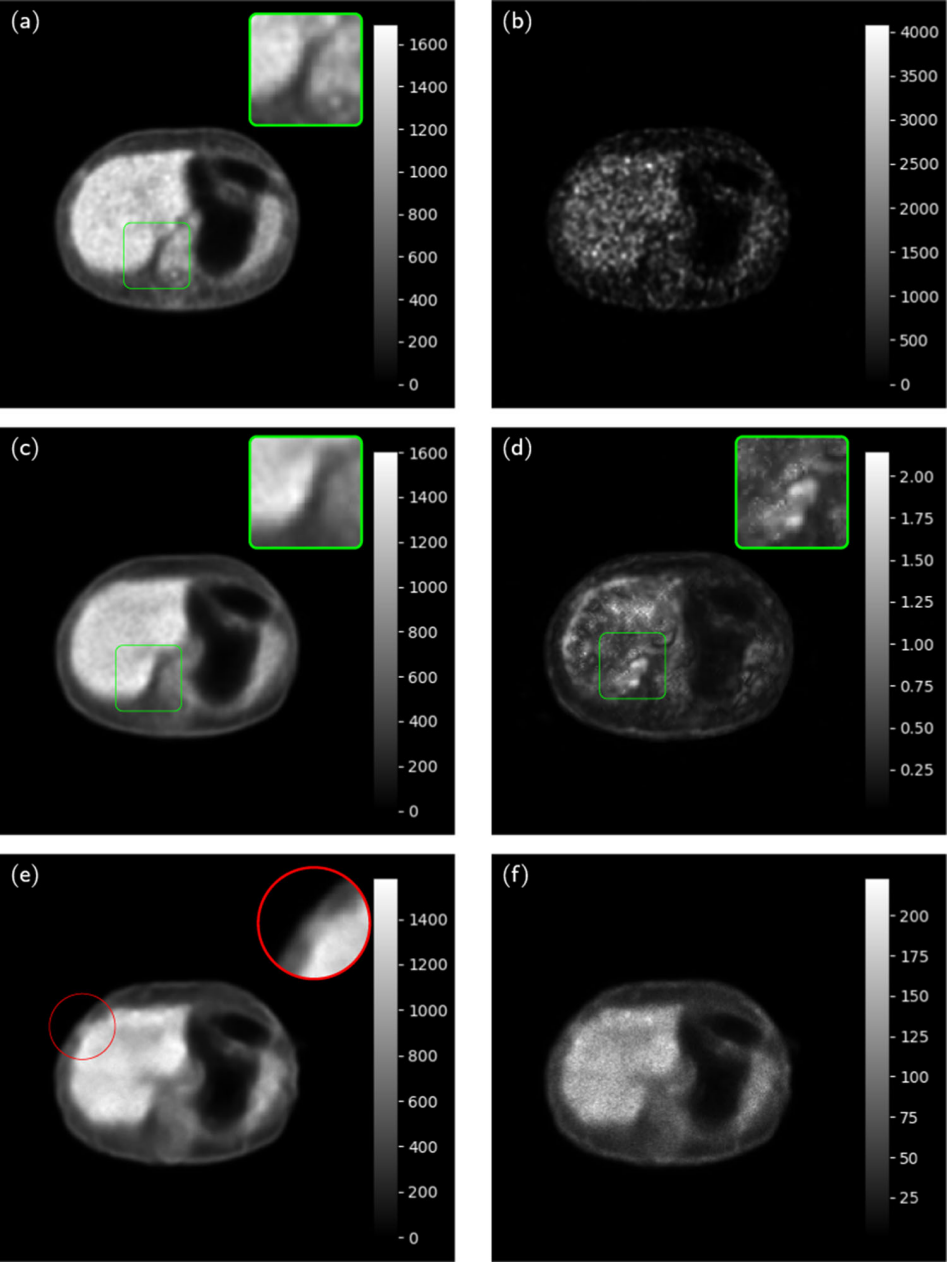
NPB-LDPET and Dropout Monte Carlo reconstructed images in comparison to the reference FD and uncertainty maps. **a**  FD reference image of the liver. **b**  LD image at DRF = 50. **c**  NPB-LDPET reconstruction.  box marks the corresponding area in the uncertainty map (**d**) that obtained higher values, as expected is most discordant with the full-dose image (**a**). The NPB-LDPET reconstructed image shows finer borders and better preservation of the structure, as compared to **e** the dropout Monte Carlo reconstructed benchmark image.  circle marks poorer borders and structure on the anterior liver (**f**) is the dropout reconstructed image uncertainty map

**Fig. 13:**
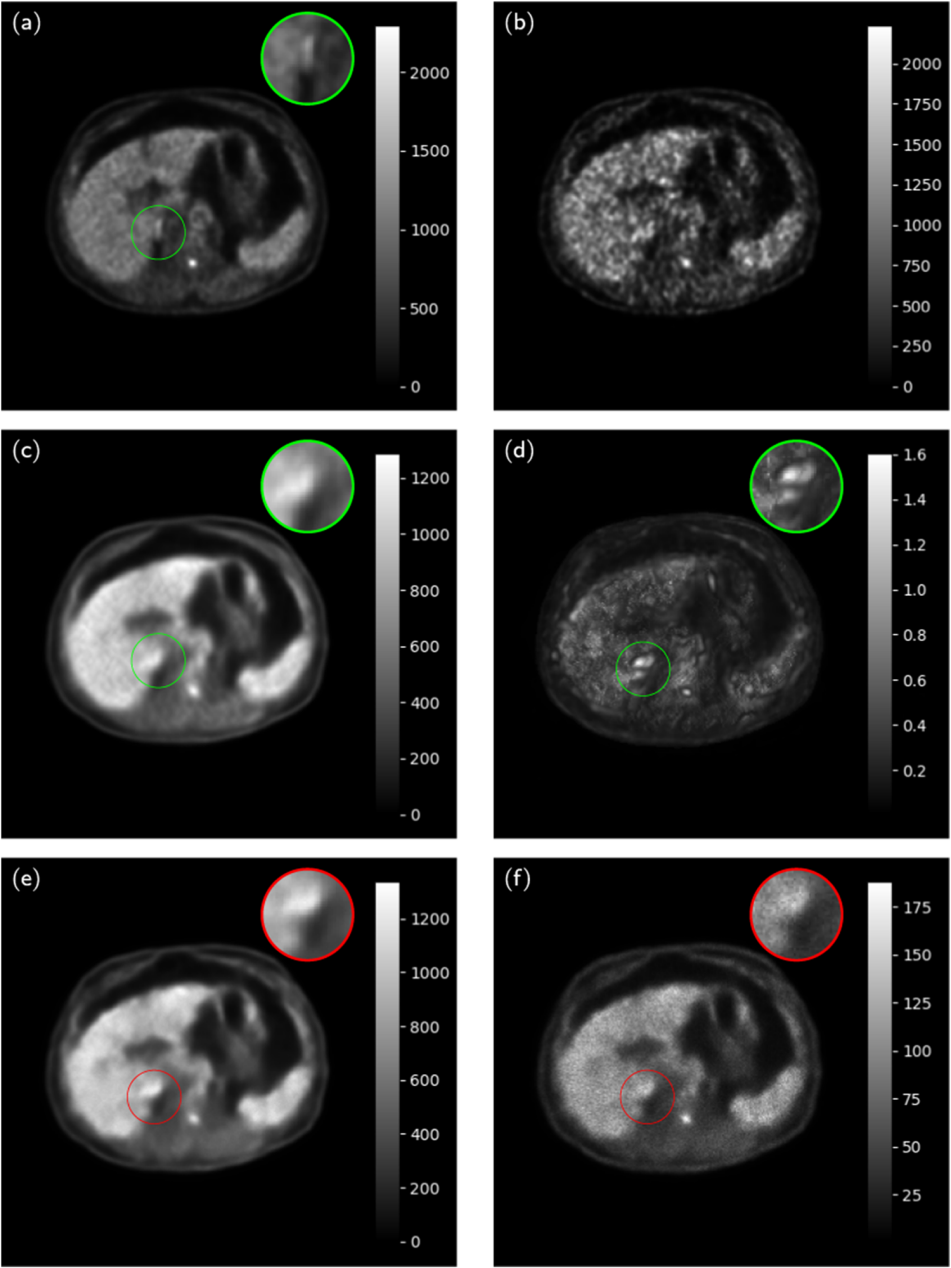
Comparison of reconstructed images using Non-Parametric Bayesian (NPB-LDPET) and Dropout methods with reference full-dose images and uncertainty maps. **a** The full-dose reference image of transverse slice of the liver.  circle show small intense focal uptake (**b**) shows a low-dose image at DRF = 50. The Bayesian LD PET reconstruction is shown in **c**, where liver foci is marked by  circle. The contrast in the liver foci is lower than that of FD, which is evident in the NPB-LDPET uncertainty map shown in **d**. The dropout reconstructed image is shown in **e** where the liver foci marked in  exhibit less contrast compared to both NPB-LDPET and FD images. **f** The dropout uncertainty map, where the same area is less noticeable

### Uncertainty map evaluation results

Visual comparisons of the NPB-LDPET and Monte Carlo FD reconstructed images and uncertainty maps were performed by an expert nuclear medicine physicist (J.K.), who has over 20 years of experience. As depicted in Figs.  12 and  13, the NPB-LDPET uncertainty maps reveal areas of discrepancy with the FD image in better clarity than the Dropout uncertainty map.

Figure 11 depicts the $r^2$ value resulting from the linear regression of the NPB-LDPET reconstruction method and Monte Carlo Dropout. The 95th percentile has an $r^2$ value of 0.9174 for the NPB-LDPET reconstruction compared to 0.6144 for the dropout Monte Carlo. The mean standard deviation score has an $r^2$ value of 0.8996 for our NPB-LDPET reconstruction method compared to 0.5977 for dropout Monte Carlo. These results demonstrate a strong correlation between both metrics and the DRF, confirming the clinical relevance of the NPB-LDPET reconstruction uncertainty map.

## Discussion and conclusions

This study introduces a non-parametric Bayesian DL model for LD PET reconstruction. To efficiently sample the posterior distribution of the DL model weights, we used the stochastic Langevin dynamics method. During inference, our approach generates multiple potential reconstructions using different sets of model weights. The final image is reconstructed by averaging the outputs from these models, and the uncertainty map is computed by evaluating a voxel-wise variance map.

Our findings suggest that the proposed NPB-LDPET framework provides a more accurate reconstruction compared to the baseline Monte Carlo dropout method for Bayesian DL, yielding enhanced lesion conspicuity and uncertainty measures that correlate more closely with the dose reduction factor. In addition to these improvements in image reconstruction, the uncertainty maps generated by our approach offer valuable insights for clinical applications. These maps highlight regions in the reconstructed images with lower reliability, particularly in areas with reduced signal-to-noise ratio, such as regions with minimal tracer uptake or low activity levels. By providing voxel-wise uncertainty estimates, these maps enable clinicians to assess the reliability of reconstructed images and make more informed diagnostic decisions. For instance, regions with high uncertainty could signal areas where image interpretation may require caution or additional validation through supplementary imaging modalities. The ability to visualize uncertainty levels in reconstructed PET images thus enhances the interpretability of DL-based reconstructions, boosting clinicians’ confidence in low-dose PET imaging results and supporting a more nuanced analysis of potential lesions or treatment responses.

The proposed approach has the potential to facilitate DL-based LD PET for clinical applications by providing vital information regarding the reliability and accuracy of the reconstructed images.
